# The anti-osteosarcoma cell activity by the sphingosine kinase 1 inhibitor SKI-V

**DOI:** 10.1038/s41420-022-00838-4

**Published:** 2022-02-03

**Authors:** Xu Sun, Hua-jian Shan, Gang Yin, Xiang-yang Zhang, Yu-min Huang, Hai-jun Li

**Affiliations:** 1 Department of Hand and Foot Surgery, Hospital Affiliated 5 to Nantong University, Taizhou People’s Hospital, Taizhou, China; 2 Department of Orthopaedics, the Second Affiliated Hospital of Soochow University, Suzhou, China; 3 Department of Orthopaedics, Wujin Hospital Affiliated to Jiangsu University, Changzhou, China; 4 Department of Orthopaedics, Tongren Hospital, Shanghai Jiao Tong University School of Medicine, Shanghai, China; 5 Department of Orthopedics, The First Affiliated Hospital of Nanjing Medical University, Nanjing, China

**Keywords:** Bone cancer, Sarcoma

## Abstract

Sphingosine kinase 1 (SphK1) expression and activity are elevated in human osteosarcoma (OS) and is a promising target of therapy. SKI-V is a non-competitive and highly-efficient non-lipid SphK1 inhibitor. The potential anti-OS cell activity by the SphK1 inhibitor was studied here. In primary OS cells and immortalized cell lines, SKI-V robustly suppressed cell survival, growth and proliferation as well as cell mobility, and inducing profound OS cell death and apoptosis. The SphK1 inhibitor was however non-cytotoxic nor pro-apoptotic in human osteoblasts. SKI-V robustly inhibited SphK1 activation and induced accumulation of ceramides, without affecting SphK1 expression in primary OS cells. The SphK1 activator K6PC-5 or sphingosine-1-phosphate partially inhibited SKI-V-induced OS cell death. We showed that SKI-V concurrently blocked Akt-mTOR activation in primary OS cells. A constitutively-active Akt1 (ca-Akt1, S473D) construct restored Akt-mTOR activation and mitigated SKI-V-mediated cytotoxicity in primary OS cells. In vivo, daily injection of SKI-V potently suppressed OS xenograft tumor growth in nude mice. In SKI-V-administrated OS xenograft tissues, SphK1 inhibition, ceramide increase and Akt-mTOR inhibition were detected. Together, SKI-V exerts significant anti-OS activity by inhibiting SphK1 and Akt-mTOR cascades in OS cells.

## Introduction

Cancer statistical studies have estimated that osteosarcoma (OS) accounts for about one-fifth of all primary bone malignancies [1, 2]. The current treatment options for OS include a three-drug (cisplatin, doxorubicin, and methotrexate) chemotherapy regimen together with surgical OS resection [3–7]. In the past decades, the five-year overall survival (70–80%) of OS have reached plateau [3–7]. A large number of OS patients can bediagnosed at advanced stages or with recurrent tumors. The prognosis of these patients is often poor, possibly due to tumor metastasis [3–7]. It is therefore important to explore the key molecular targets of OS development and progression, and to explore novel and efficient therapeutic agents [3, 7, 8].

Sphingosine kinases (SphKs), including SphK1 and SphK2, are responsible for phosphorylating sphingosine to sphingosine-1-phosphate (S1P) [9–12]. The latter, S1P, acts as a signaling lipid messenger [9–12]. S1P could promote cell proliferation and survival by acting as a intracellular second messenger [13, 14]. It could also function as the ligand for EDG1 (endothelial differentiation gene 1) [9–12]. SphK1 overexpression and activation could increase cellular S1P contents, thereby promoting cancer initiation and progression [9–12]. Conversely, SphK1 inhibition (by its inhibitors), silencing (using genetic measures), or loss-of-function of mutations shall lead to depleted S1P formation, while inducing accumulation of pro-apoptotic ceramide and sphingosine [9–12], eventually causing growth arrest and apoptosis in cancer cells [9–12].

SphK1 expression is significantly elevated in clinical OS specimens [15]. Recent studies have supported that targeting SphK1 could produce significant anti-OS activity. Wei et al. showed that furowanin A inhibited SphK1 to exert significant anti-proliferative and pro-apoptotic activities in OS cells [16]. Phenoxodiol and doxorubicin co-treatment synergistically inhibited SphK1, suppressing OS cell growth [17]. Yao et al. showed that microRNA-3677 (miR-3677) silenced its target SphK1 to arrest OS cell growth [15]. Zhou et al. also found that microRNA-124, by silencing SphK1, suppressed the proliferation and invasion of OS cells [18].

SKI-V is a non-competitive, potent, and non-lipid SphK1 small molecule inhibitor with an IC_50_ of 2 μM [19, 20]. It inhibited SphK1 activity and S1P contents, causing apoptosis in T24 bladder cancer cells [20]. Intraperitoneal injection of SKI-V significantly inhibited mammary adenocarcinoma xenograft growth in immunocompetent BALB/c mice [20]. It however failed to induce immediate or delayed toxicity at doses up to 75 mg/kg in Swiss-Webster mice and BALB/c nude mice [20]. We here tested its potential anti-cancer effect in OS cells.

## Results

### SKI-V exerts significant anti-OS cell activity

**Fig. 1:**
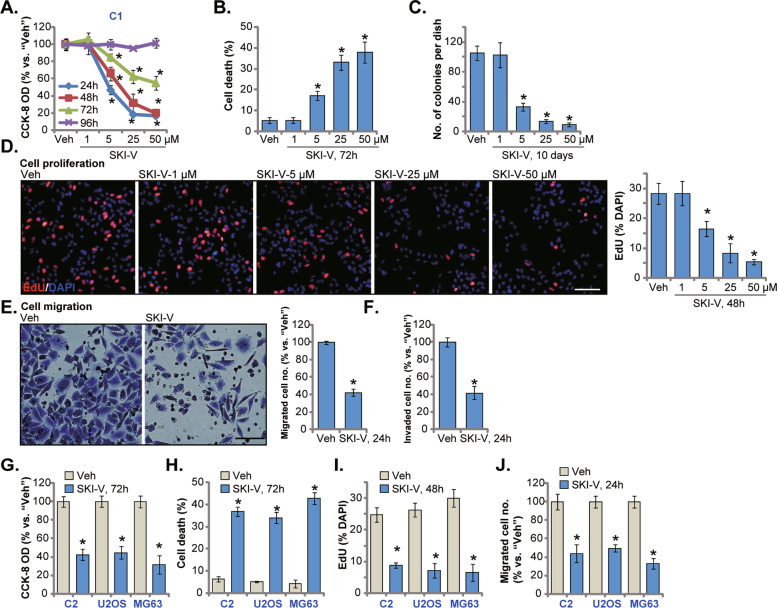
SKI-V exerts significant anti-OS cell activity. The primary human OS cells (“C1”/“C2”, **A**–**J**) or the immortalized cell lines (U2OS and MG-63, **G**–**J**) were cultured in FBS-containing compete medium and treated with SKI-V (at 25 μM or indicated concentrations) for applied time periods; Cellular functions, including cell viability (**A** and **G**), cell death (Trypan blue staining assays, **B** and **H**), colony formation (**C**), cell proliferation (EdU assays, **D** and **I**), as well as cell migration (“Transwell” assays, **E** and **J**) and invasion (“Matrigel Transwell” assays, **F**) were tested by the appropriate assays mentioned, with results quantified “Veh” stands for the vehicle control. Data were presented as mean ± standard deviation (SD, *n* = 5). *P < 0.05 vs. “Veh” group. Experiments were repeated five times with similar results obtained. Scale bar = 100 μm (**D**, **E**).

C1 primary OS cells were cultivated in complete medium and treated with SKI-V (from 1 to 50 μM). As shown SKI-V decreased the viability of C1 primary OS cells in a concentration-dependent manner (Fig. 1A). At 5–50 μM, the SphK1 inhibitor significantly decreased the CCK-8 OD (Fig. 1A). At 1 μM SKI-V was ineffective (Fig. 1A). In addition, SKI-V required 48 h to exert a significant activity in C1 OS cells, showing a time-dependent response (Fig. 1A). Moreover, SKI-V-induced viability reduction in C1 primary cells lasted for 96 h (Fig. 1A). By employing Trypan blue staining assays, we showed that SKI-V dose-dependently induced C1 cell death (Fig. 1B). Furthermore, treatment with the SphK1 inhibitor (5–50 μM) largely inhibit the number of viable C1 cell colonies (results quantified in Fig. 1C), further supporting the cytotoxic activity of SKI-V against primary OS cells.

Next, nuclear EdU staining assays were carried out. Results showed that SKI-V, at 5–50 μM, potently decreased the ratio of EdU-stained nuclei in C1 primary cells (Fig. 1D), supporting its anti-proliferative activity. As shown, 25 μM of SKI-V led to significant viability reduction (Fig. 1A), cell death (Fig. 1B), decreased colony formation (Fig. 1C), and proliferation inhibition (Fig. 1D) in C1 OS cells. This concentration was therefore selected for the following studies. Treatment with SKI-V (25 μM) potently suppressed C1 primary cell migration and invasion in vitro, examined through “Transwell” and “Matrigel Transwell” assays (Fig. 1E, F), respectively.

We also tested whether SKI-V could exert similar activity in other OS cells. C2 OS cells (derived from another primary OS patient) and the immortalized OS cell lines (U2OS and MG-63) were cultivated in complete medium and treated with SKI-V at 25 μM. The SphK1 inhibitor potently decreased viability (CCK-8 OD) in the immortalized and primary OS cells (Fig. 1G). Increased Trypan blue staining, indicating cell death, was observed in the SKI-V-treated primary and immortalized OS cells (Fig. 1H). Moreover, SKI-V robustly suppressed proliferation (tested by reduction of the EdU-stained nuclei ratio, Fig. 1I) and migration (quantification from the “Transwell” assays, Fig. 1J) in the OS cells. Thus SKI-V exerted robust anti-OS cell activity.

### Apoptosis activation in SKI-V-treated OS cells

**Fig. 2:**
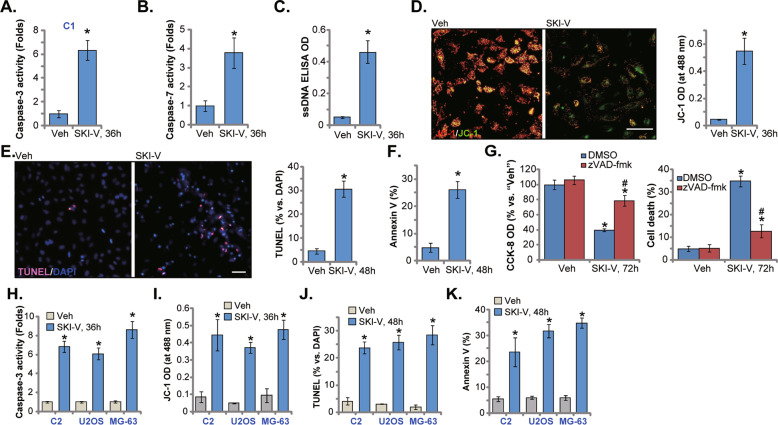
Apoptosis activation in SKI-V-treated OS cells. The primary human OS cells (“C1”/“C2”, **A**–**F**, **H**–**K**) or the immortalized cell lines (U2OS and MG-63, **H**–**K**) were cultured in FBS-containing compete medium and treated with SKI-V (at 25 μM) for applied time periods; The relative caspase-3 and caspase-7 activities (**A**, **B** and **H**), single strand DNA contents (ELISA OD, **C**), mitochondrial depolarization (JC-1 staining assays, **D** and **I**) were tested; Cell apoptosis was tested by nuclear TUNEL staining (**E** and **J**) and Annexin V-PI FACS (**F** and **K**) assays, with results quantified. The C1 primary OS cells were pretreated with the pan caspase inhibitor z-VAD-fmk (50 μM) or 0.25% DMSO for 30 min, followed by SKI-V (at 25 μM) treatment for 72 h, cell viability and death were examined by CCK-8 and Trypan blue staining assays, respectively (**G**). “Veh” stands for the vehicle control. Data were presented as mean ± standard deviation (SD, *n* = 5). *P < 0.05 vs. “Veh” group. ^#^*P* < 0.05 vs. DMSO group (**G**). Experiments were repeated five times with similar results obtained. Scale bar = 100 μm (**D** and **E**).

SphK1 inhibition will led to ceramide production to promote cancer cell apoptosis [14, 21–24]. As shown, the caspase-3/-7 activities were robustly augmented in SKI-V (25 μM)-treated C1 primary OS cells (Fig. 2A, B). ssDNA contents were significantly increased after SKI-V (25 μM) treatment, indicating enhanced DNA breaks (Fig. 2C). Further studies demonstrated that SKI-V-induced mitochondrial membrane potential (MMP) collapse and mitochondrial depolarization in C1 primary OS cells, causing accumulation of the JC-1 green monomers (Fig. 2D). Apoptosis was induced in SKI-V (25 μM)-treated C1 primary OS cells, as the ratio of TUNEL-stained nuclei was increased significantly (Fig. 2E). Apoptosis activation was also confirmed by the fact that the Annexin V-positive C1 OS cells were significantly increased after SKI-V treatment (Fig. 2F).

Significantly SKI-V-induced C1 OS cell viability reduction (Fig. 2G) and cell death (Fig. 2G) were largely ameliorated by z-VAD-fmk, the pan caspase inhibitor. In C2 cells and immortalized cell lines (U2OS and MG-63), SKI-V augmented caspase-3 activity (Fig. 2H) and induced significant mitochondrial depolarization (evidenced by the increased JC-1 green monomers, Fig. 2I). Treatment with SKI-V provoked apoptosis in the OS cells as well, as the ratio of TUNEL-stained nuclei (Fig. 2J) and the number of the Annexin V-stained cells (Fig. 2K) were both significantly augmented. Thus, SKI-V provoked OS cell apoptosis.

### The effect of SKI-V in hFOB1.19 human osteoblastic cells and primary osteoblasts

**Fig. 3:**
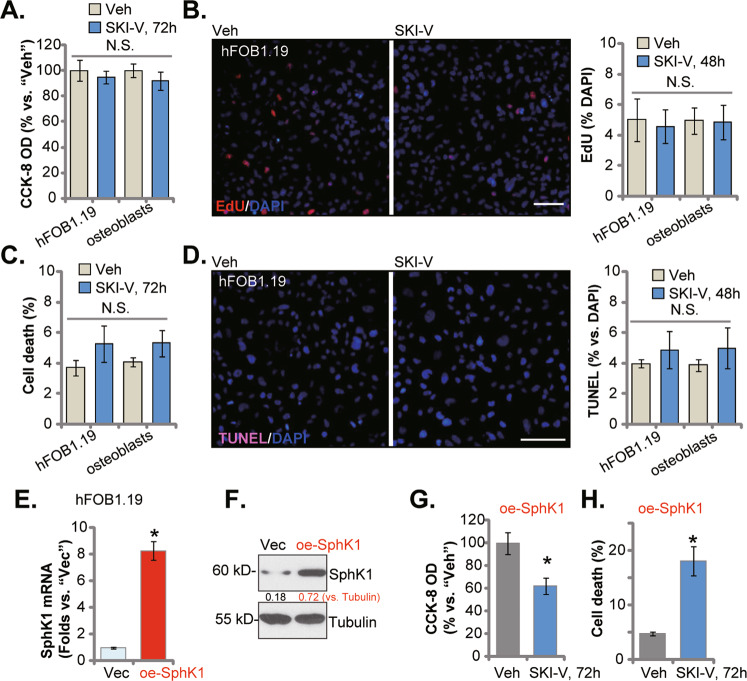
The effect of SKI-V in hFOB1.19 human osteoblastic cells and primary osteoblasts. The hFOB1.19 human osteoblastic cells or the primary human osteoblasts (“osteoblasts”) were cultured and treated with SKI-V (25 μM) for applied time periods; cell viability, proliferation, death, and apoptosis were tested by CCK-8 (**A**), nuclear EdU staining (**B**), trypan blue staining (**C**) and nuclear TUNEL staining (**D**) assays, respectively. “Veh” stands for the vehicle control. Data were presented as mean ± standard deviation (SD, *n* = 5). Expression of the *SphK1 mRNA* (**E**) and protein (**F**) in the stable hFOB1.19 cells with the SphK1-expressing lentiviral construct (“oe-SphK1”) or the empty vector (“Vec”) was shown. The oe-SphK1 hFOB1.19 cells were treated with SKI-V (25 μM) or vehicle control for 72 h; cell viability and death were tested by CCK-8 (**G**) and trypan blue staining (**H**) assays, respectively. “N. S.” stands for non-statistical difference (*P* > 0.05). *P < 0.05 *vs*. “Vec”/“Veh” group. Experiments were repeated five times with similar results obtained. Scale bar = 100 μm (**B** and **D**).

The potential effect of SKI-V in non-cancerous osteoblasts was examined next. As described, hFOB1.19 osteoblastic cells and the primary human osteoblasts (“osteoblasts”) were cultured and treated with SKI-V (25 μM). As demonstrated, SKI-V did not significantly decrease the viability (CCK-8 OD) in hFOB1.19 osteoblastic cells and primary osteoblasts (Fig. 3A). Moreover, the ratio of EdU-stained nuclei was not significantly altered in SKI-V-treated hFOB1.19 osteoblastic cells and primary osteoblasts (Fig. 3B). In addition, results from Trypan blue staining assay (Fig. 3C) and nuclear TUNEL staining assay (Fig. 3D) showed that SKI-V (25 μM) did not induce significant cell death and apoptosis in hFOB1.19 cells and primary osteoblasts.

Next a SphK1-expressing lentiviral construct [15] was transduced to hFOB1.19 osteoblastic cells, and the stable cells were established after selection through the puromycin-containing medium (namely “oe-SphK1” cells). The *SphK1* mRNA and protein expression were significantly increased in the oe-SphK1 hFOB1.19 cells (Fig. 3E, F). Importantly, treatment with SKI-V (at 25 μM) significantly inhibited viability (Fig. 3G) and induced cell death (tested by the Trypan blue staining assays, Fig. 3H) in oe-SphK1 hFOB1.19 cells.

### SphK1 inhibition in SKI-V-treated OS cells

**Fig. 4:**
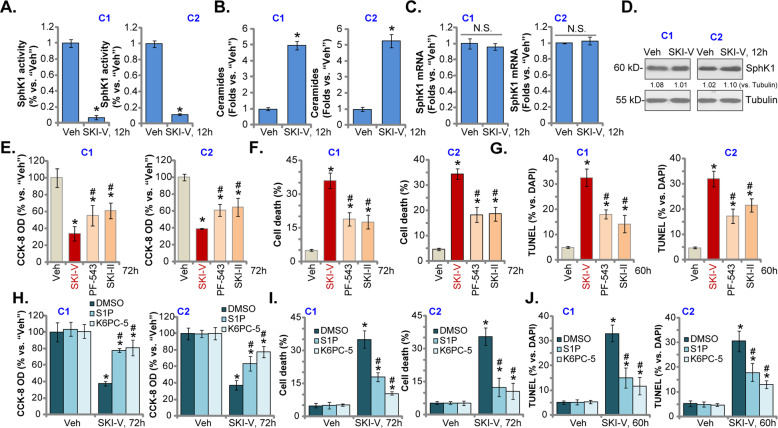
SphK1 inhibition in SKI-V-treated OS cells. The primary OS cells (“C1”/“C2”) were cultured in FBS-containing complete medium and treated with SKI-V (at 25 μM) for applied time periods, the relative SphK1 activity (**A**) and ceramide contents (**B**) were shown; expression of *SphK1* mRNA and protein was tested by qRT-PCR (**C**) and western blotting (**D**) assays. “C1” and “C2” primary cells were cultured in FBS-containing compete medium and treated with 25 μM of SKI-V, SKI-II or PF-543 for applied time periods, cell viability, death, and apoptosis were tested by CCK-8 (**E**), trypan blue staining (**F**) and nuclear TUNEL staining (**G**) assays, respectively. The primary human OS cells (“C1”/“C2”) were pretreated with K6PC-5 (20 μM), S1P (20 μM) or vehicle control (0.1% DMSO), followed by SKI-V (at 25 μM) treatment for 60 h/72 h, cell viability, death, and apoptosis were examined by CCK-8 (**H**) and trypan blue staining (**I**) and nuclear TUNEL staining (**J**) assays, respectively. “Veh” stands for the vehicle control. Data were presented as mean ± standard deviation (SD, *n* = 5). *P < 0.05 vs. “Veh”. ^#^*P* < 0.05 *vs*. SKI-V group (**E**–**G**). ^#^*P* < 0.05 *vs*. “DMSO” group (**H**–**J**). “N. S.” stands for non-statistical difference (*P* > 0.05). Experiments in this Figure were repeated five times with similar results obtained.

Since SKI-V is a SphK1 inhibitor. As shown, in primary OS cells, C1 and C2, treatment with SKI-V (25 μM) led to 80–90% inhibition of SphK1 activity (Fig. 4A). Conversely, the cellular ceramide contents increased over 4–5 folds (Fig. 4B). Notably, the *SphK1* mRNA/protein (Fig. 4C, D) expression was not significantly altered by the SKI-V in the primary OS cells.

We next compared the activity of SKI-V with other established SphK1 inhibitors, SKI-II [25–27] and PF-543 [28, 29]. As shown, at the same concentration (25 μM) SKI-V-induced CCK-8 viability reduction (Fig. 4E), cell death (Fig. 4F) and apoptosis (evidenced by the TUNEL-stained nuclei ratio increasing, Fig. 4G) were more potent than both SKI-II and PF-543. These results indicated that there could be SphK1-independent mechanisms underlying SKI-V-induced actions in OS cells. K6PC-5, a SphK1 activator [30–33], and S1P were utilized. As shown, in C1 and C2 primary OS cells, pretreatment with K6PC-5 or S1P attenuated, but not reversed, SKI-V-induced viability reduction (Fig. 4H), cytotoxicity (Trypan blue assays, Fig. 4I), and apoptosis (TUNEL assays, Fig. 4J).

### Akt-mTOR inhibition in SKI-V-treated OS cells

**Fig. 5:**
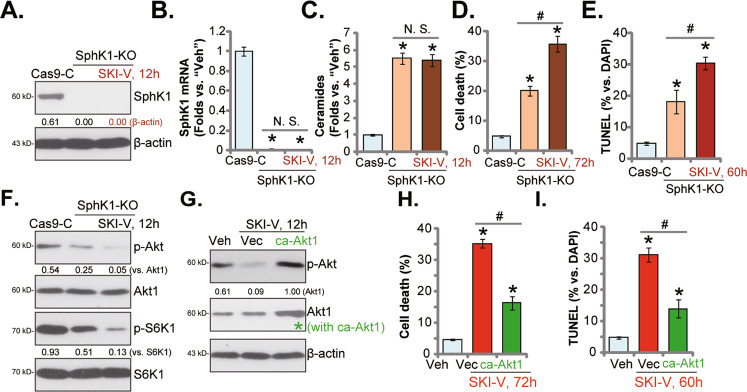
Akt-mTOR inhibition in SKI-V-treated OS cells. The primary OS cells (“C1”), stably expressing a CRISPR/Cas9-SphK1 KO construct (“SphK1-KO” cells), were treated with or without 25 μM of SKI-V, and cultured for applied time periods; control cells were stably expressing the control CRISPR/Cas9 vector (“Vec”); expression of listed proteins and mRNA were tested by western blotting (**A** and **F**) and qRT-PCR (**B**) assays. Cellular ceramide contents were examined (**C**); cell death and apoptosis were tested by trypan blue staining (**D**) and nuclear TUNEL staining assays (**E**), respectively, with results quantified. The primary OS cells (“C1”), stably expressing the constitutively-active Akt1 (ca-Akt1, S473D) or empty vector (“Vec”) were treated with 25 μM of SKI-V for applied time periods; expression of listed proteins was tested by Western blotting assays (**G**). Cell death (Trypan blue staining assays, **H**) and apoptosis (by measuring TUNEL-stained nuclei ratio, **I**) were tested. “Veh” stands for the vehicle control. Data were presented as mean ± standard deviation (SD, *n* = 5). *P < 0.05 vs. “Cas9-C”/“Veh” cells. ^**#**^*P* < 0.05 (**D**, **E**, **H** and **I**). “N. S.” stands for non-statistical difference (*P* > 0.05) (**B** and **C**). Experiments in this Figure were repeated five times with similar results obtained.

A CRISPR/Cas9-SphK1-KO construct [15] was applied to stably knockout (KO) SphK1 in C1 primary OS cells (“SphK1-KO” cells) (Fig. 5A, B). As shown in C1 primary cells SphK1 KO induced significant ceramide production (Fig. 5C), cell death (Fig. 5D), and apoptosis (evidenced by the TUNEL-stained nuclei ratio increasing, Fig. 5E). Importantly, although SKI-V treatment did not affect SphK1 expression (Fig. 5A, B) and ceramide production in SphK1-KO cells (Fig. 5C), it did induce further cytotoxicity by enhancing cell death (Fig. 5D) and apoptosis (by measuring the ratio of the TUNEL-stained nuclei, Fig. 5E). Thus, SKI-V was still cytotoxic in SphK1-KO OS cells, further supporting the existence of SphK1-independent mechanisms for SKI-V-induced anti-OS cell activity.

Studies have proposed that SKI-V could also target PI3K-Akt cascade [19, 20]. Although CRISPR/Cas9-induced SphK1 KO inhibited Akt-S6K1 phosphorylation in C1 primary OS cells (Fig. 5F), adding SKI-V resulted in further Akt-S6K1 inhibition (Fig. 5F). Therefore, besides SphK1 inhibition, SKI-V could further inhibit Akt-mTOR cascade (Fig. 5F). Next, to C1 primary OS cells a constitutively-active Akt1 (“caAkt1”, S473D) viral construct was stably transduced (see the “green star” in Fig. 5G), that completely restored Akt phosphorylation in SKI-V-treated cells (Fig. 5G). Importantly, caAkt1 largely attenuated SKI-V-induced cell death and apoptosis (Fig. 5H, I) in C1 primary OS cells. Thus, concurrent inhibition of both SphK1 and Akt-mTOR cascades by SKI-V resulted in profound OS cell death and apoptosis.

### The anti-OS cell activity of SKI-V in vivo

**Fig. 6:**
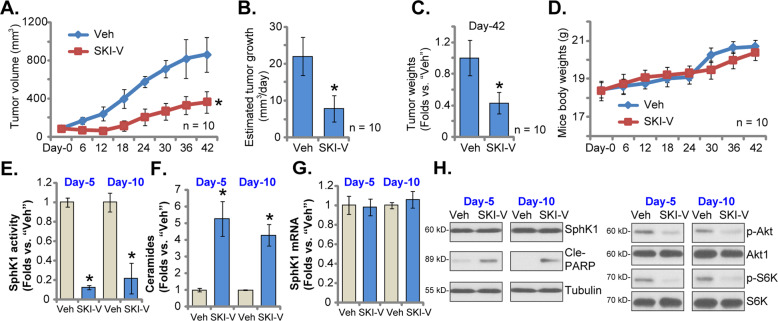
The anti-OS cell activity of SKI-V in vivo. The “C1” OS xenograft-bearing nude mice were subject to intraperitoneal (*i.p*.) injection of SKI-V (30 mg/kg body weight, daily for 18 days) or vehicle control (“Veh”), with ten mice per group. Tumor volumes (**A**) and mice body weights (**D**) were recorded every six days (from Day-0 to Day-36). The estimated daily tumor growth, in mm^3^ per day, was calculated (**B**). At Day-36, all mice were anaesthetized and decapitated, OS xenografts were weighted (**C**). At experimental Day-5 and Day-10, one tumor of each group was isolated (total for tumors), the relative SphK1 activity (**E**), ceramide contents (**F**), and expression of *SphK1* mRNA (**G**) and listed proteins (**H**) were shown. Data were presented as mean ± standard deviation (SD). *P < 0.05 vs. “Veh” group.

At last the C1 primary OS cells were *s.c*. injected to the flanks of nude mice. After 20 days, OS xenografts (100 mm^3^ per tumor) were established (as “Day-0”). The xenograft-bearing nude mice were then assigned into two different groups. The treatment group received intraperitoneal (*i.p*.) injection of SKI-V (at 30 mg/kg body weight, daily administration for 18 consecutive days). The other group received vehicle control administration (“Veh”). The tumor growth curve, recording tumor volumes every six days, showed that daily SKI-V injection robustly suppressed OS xenograft growth in nude mice (Fig. 6A). The volumes of OS xenografts with SKI-V injection were dramatically lower than those of vehicle administration (Fig. 6A). The formula, (Tumor volume at Day-36 subtracting tumor volume at Day-0)/36, was utilized to calculate the estimated daily tumor growth was calculated under. Results showed that the growth of OS xenografts in the nude mice was largely inhibited following SKI-V injection. At Day-36, all mice were anaesthetized and decapitated, OS xenografts were isolated carefully and weighted. OS xenografts with SKI-V injection were significantly lighter than the xenografts with the vehicle control treatment (Fig. 6C). The mice body weights were however not significantly different among the two mice groups (Fig. 6D). No significant animal toxicities, including fever, vomiting, hair loss, and neurological symptoms, were detected in the SKI-V-treated nude mice.

At the experimental Day-5 and the experimental Day-10, one tumor of each group was isolated. Tissue lysates of the four isolated OS xenografts were analyzed. As shown, the SphK1 activity was significantly decreased in SKI-V-treated OS xenograft tissues (Fig. 6E). Conversely, increased ceramide contents were detected (Fig. 6F). *SphK1* mRNA/protein expression (Fig. 6G, H) in tumor tissues was unchanged with SKI-V administration. Levels of phosphorylated-Akt (Ser-473) and phosphorylated-S6K1 were significantly inhibited in SKI-V-treated OS xenograft tissues (Fig. 6H), where levels of cleaved PARP were significantly increased (Fig. 6H). These results suggested that SKI-V administration induced SphK1 inhibition, ceramide accumulation, Akt-mTOR inactivation, and apoptosis induction in OS xenografts.

## Discussion

Three conventional OS subtypes are recognized, including osteoblastic, chondroblastic and fibroblastic OS [34, 35]. Recent molecular profiling studies together with exploring the tissue banks have led to an increased understanding for the biology and pathological mechanisms of OS progression [3, 7, 8]. Yet, the clinical outcomes for the advanced OS is still far from satisfactory, with a poor median survival time particularly in patients with metastatic or recurrent OS [3, 7, 8]. It is therefore urgent to explore new therapeutic agents that effectively target OS [3, 7, 8].

Pharmacological or genetic means were applied to silence or inhibit SphK1, inhibiting OS cell growth and inducing cell apoptosis [15–18]. SKI-V is a non-competitive and highly-efficient SphK1 inhibitor. SKI-V intraperitoneal injection largely suppressed mammary adenocarcinoma xenograft growth in mice [20]. Gong et al. found that SKI-V facilitated bortezomib-induced ceramide production and apoptosis in pancreatic cancer cells [36].

We showed that SKI-V exerted significant anti-tumor activity in OS cells. In the primary and immortalized OS cells, treatment with the SphK1 inhibitor inhibited cell survival, growth, proliferation and cell mobility, and inducing profound cell death and apoptosis. It however failed to significant cytotoxicity in human osteoblasts. SKI-V inhibited SphK1 activation and induced ceramide accumulation, without affecting SphK1 expression in primary human OS cells. The SphK1 activator K6PC-5 or S1P partially inhibited SKI-V-induced OS cell death and apoptosis. In vivo, daily injection of SKI-V robustly suppressed OS xenograft tumor growth. The experimental mice were well-tolerated to the treatment regimen, as no significant toxicities were reported. In SKI-V-treated OS xenograft tissues, SphK1 inhibition, ceramide increase, and apoptosis activation marker were detected.

Due to genetic mutations (PI3KCA, PTEN depletion, and receptor tyrosine kinase activation, etc) [37, 38], Akt-mTOR cascade is frequently hyper-activated in OS, contributing to tumor initiation and disease progression [7, 37–42]. This pathway is important for OS cell proliferation, migration, cell cycle progression, apoptosis resistance, angiogenesis, metastasis, and therapy resistance [39, 40]. Conversely, Akt-mTOR inhibition using small molecule inhibitors or genetic methods could result in significant anti-OS cell activity, represents as a promising therapeutic approach [39, 40]. Zhu et al. have shown that XL3388 blocked mTORC1/2 activation and inhibited OS cell growth [43]. Liu et al. have shown that celecoxib induced apoptosis activation in MG-63 cells via downregulation of PI3K-Akt signaling [44]. Jin et al. found that PI3K-Akt signaling inactivation by grifolin induced OS cell apoptosis [45].

We found that SKI-V inhibited Akt-mTOR activation in primary human OS cells, an effect that was parallel to SphK1 inhibition. Akt-mTOR inactivation was observed as well in OS xenograft tumor tissues after SKI-V injection. The caAkt1 restored Akt-mTOR activation and ameliorated SKI-V-induced OS cell death. Therefore, concurrent inhibition of Akt-mTOR cascade by the SphK1 inhibitor could explain its superior anti-OS activity. Indeed, SKI-V-induced cytotoxicity against primary human OS cells was significantly more potent than two other established SphK1 inhibitors (PF-543 and SKI-II).

## Conclusion

SKI-V exerts significant anti-OS activity by targeting SphK1 and Akt-mTOR cascades in OS cells.

## Materials and methods

### Chemicals, reagents, and antibodies

Puromycin, polybrene, cell counting kit-8 (CCK-8), z-VAD-fmk were provided by Sigma-Aldrich (St. Louis, MO). Antibodies for p70 S6 Kinase (S6K1, Thr389, #9205), S6K1 (#9202), phosphorylated-Akt Ser-473 (#9271) and Akt1 (#9272), SphK1 (#12071), SphK2 (#32346), β-Tubulin (#2146) and β-actin (#3700) were purchased from Cell Signaling Technologies (Beverly, MA). JC-1, DAPI, Lipofectamine 3000, TUNEL (terminal deoxynucleotidyl transferase dUTP nick end labeling) apoptosis assay kit, Annexin V-propidium iodide (PI) FACS kit were provided by Thermo-Fisher Invitrogen (Carlsbad, CA). SKI-V, SKI-II, PF-543, and S1P were provided by Selleck (Shanghai, China).

### Cell culture

U2OS and MG63, as well as the primary human OS cells (from two written-informed consent primary patients, “C1/C2”), were from Dr. Cao [46, 47]. The primary human osteoblasts and hFOB1.19 osteoblastic cells were from Dr. Ji [48], and cells cultivated under the described protocols [49, 50]. The protocols using human cells were approved the Ethic Committee of Taizhou People’s Hospital, according with the principles expressed in the Declaration of Helsinki.

### Other cellular function studies

Cells were seeded into ploy-L-lysine-coated tissue-culturing plates at optimal seeding density, and the detailed protocols of the cellular functional assays, including CCK-8 viability, Trypan blue staining, colony formation, EdU (5-ethynyl-20-deoxyuridine) staining, and the in vitro cell migration and migration (“Transwell” assays) as well as Annexin V FACS, TUNEL staining, mitochondrial depolarization JC-1 staining and single strand DNA (ssDNA) ELISA were described in the previous studies [51, 52].

### Quantitative real time-PCR (qRT-PCR) and Western blotting assays

The detailed protocols for qRT-PCR and Western blotting assays were described in detail elsewhere [46, 47].

### Caspase-3/-7 activity assay

Following treatment, 30 μg protein lysates per treatment were dissolved in the caspase assay buffer and were incubated with the corresponding 7-amino-4-trifluoromethylcoumarin (AFC)-conjugated caspase-3/-7 substrate. After 3 h, an Infinite 200 PRO reader was employed to examine AFC activity.

### SphK1 activity assay

After the designated treatments, cells and tissues were homogenized and centrifuged at 12,000 rpm to obtain the supernatant. SphK1 activity was determined by a SphK1 activity assay kit (Abnova). The attached SphK1 substrate was added to the supernatant (25 μL per treatment) for 5 min at 30 °C, and the SphK1 activity was detected by a microplate reader.

### Ceramide assay

The detailed protocols of analyzing cellular ceramides were described previously [36]. Ceramides were expressed as fmol in nmol of phospholipid and were always normalized to that of control treatment.

### CRISPR/Cas9-induced knockout of SphK1

OS cells were first stably transduced with the Cas9-expressing construct (Genechem, Shanghai, China). Cells were further transfected with a lentiCRISPR SphK1-KO plasmid (from Dr. Yao [15]), and thereafter the single stable cells were established after puromycin selection and PCR-mediated screening of SphK1 KO.

### Constitutively-active mutant Akt1

The constitutively-active Akt1 (“caAkt1”, S473D) adenoviral construct was from Dr. Liu [21]. The adenovirus was directly added to the cultured OS cells. The caAkt1 expression was always verified by Western blotting in puromycin-selected stable cells.

### SphK1 overexpression

A GV369 SphK1-expressing lentiviral construct [15] was transduced to the hFOB1.19 cells, and the stable cells were established with puromycin selection, with SphK1 overexpression verified regularly.

### OS xenograft studies

The animal procedures were conformed to the Ethics Committee and Animal Care Committee of Taizhou People’s Hospital (Taizhou, China). The five-six week-old BALB/c nude mice (half male half female) were provided by Shanghai Slake Laboratory Animal Co. (Shanghai, China). The nude mice were maintained in controlled standard environmental conditions. The C1 primary human OS cells (at six million cells per mouse, in 200 µL serum-free DMEM/Matrigel) were subcutaneously (*s.c*.) injected into the flanks of nude mice. After 20 days of cell injection, OS xenografts were established. The xenograft-bearing nude mice were subject to intraperitoneal (i.p.) injection of SKI-V (30 mg/kg body weight, daily for 18 consecutive days) or the vehicle control. The volume of each xenograft tumor was calculated using the following formula: π/6 × *L* (long diameter) × *W* (short diameter)^2^.

### Statistical analyses

Data with normal distributions were presented as mean ± standard deviation (SD). The significance between groups were determined by the two-tailed Student’s t test (Excel 2007, for two groups) or ANOVA analysis and Student-Newman-Keuls post hoc test (SPSS 23.0, for multiple groups). *P* values < 0.05 were considered as statistically significant.

## Data Availability

All data are available upon request.
